# Supplementary material for: Evidence for a constant occipital spotlight of attention using MVPA on EEG data
Source: PLoS One. 2025 Mar 26;20(3):e0320233. doi: 10.1371/journal.pone.0320233 (PMC11940686; doi:10.1371/journal.pone.0320233)
Supplement: Attachment — Submitted filename: Reviewer_response.docx [file pone.0320233.s002.docx]

Dear Dr. Corsi,

We would like to thank you and the reviewer for your valuable time and for your helpful and constructive comments, which have been of great help in improving this work.

In the revised version of the manuscript, we have basically addressed all suggestions, including 1) simulated data to relate theoretical models to empirical data, 2) cross-correlation between the ERP lateralization index/alpha band activity and the classification accuracy to ensure a more robust interpretation of the data, and a clarification of 3) the reason for emphasizing the distinction between conventional analysis and MVPA, 4) why our methodology of analysis is unfortunately incompatible with the collection of useful behavioural data, and 5) the minor comments. The only point that has not resulted in any change in the manuscript is the one related to the whole-scalp decoding. We clarify here the explanation on the occipital channels selection, but we also provide whole-scalp decoding results, proving that it is more appropriate to keep the manuscript with the initial selection of occipital electrodes guided by our hypotheses.

A detailed explanation of how we have addressed the reviewer’s concerns has been appended in this letter, with the original reviewer’s comments in bold and our responses below. The changes made in the revised manuscript have been highlighted in blue to facilitate the review.

Yours sincerely,

María Melcón and Simon Hanslmayr

(on behalf of the co-authors)

**REVIEWERS' COMMENTS:**

**Reviewer #1: Review of Evidence for constant occipital spotlight of attention using MVPA on EEG data

This study investigates the temporal dynamics of visuospatial attention, challenging the traditional view that attention remains fixed at a single location. Using EEG-based classification and conventional analyses, the authors examined how attention fluctuates over space and time during visuospatial cueing tasks with central and peripheral cues.

While this paper presents some interesting aspects, including the great practice of code sharing, I believe substantial revisions are necessary before it can be recommended for publication. The authors take an interesting approach by using multiple datasets and analytical methods to address their research question. However, many of the conclusions appear to rely on observational inference, which does not provide sufficient empirical support.**

Response: Thank you very much for both the positive comments and the feedback. We have tried to clarify the aspects pointed out in the review to improve the manuscript. 
 **The primary issue, in my opinion, lies in the authors' effort to propose different theoretical models to interpret their results—an estimable practice —but the integration of these models with the actual findings lacks rigorous evidence. I suggest the authors explore methodologies from the field of Representational Similarity Analysis (RSA), where theoretical models are directly correlated with empirical data. This approach allows researchers to determine which model aligns most closely with the observed patterns, providing a stronger basis for their claims. Currently, the assertion that one experiment reveals a specific pattern over another relies too heavily on the subjective observation of "visually similar" results.**

Response: we greatly appreciate this recommendation, as this suggestion can help us to make strong statements in the interpretation of our data. In order to integrate the results from the classification analyses with the conventional analyses, we simulated two datasets containing modulations in the electrophysiological correlates of attention, i.e., the P1/N1 complex and the alpha band. Our results show that the peripheral cue dataset fits better with the ERP than the alpha simulation and, at the same time, the ERP simulation aligns most closely with the peripheral cue dataset.

First, we have included a new paragraph in the introduction to present these analyses (page 5):

“Eventually, we implemented the opposite approach to identify the TGM pattern to expect under the modulations of the electrophysiological attentional correlates, that is, ERPs and alpha activity. Thus, we simulated two datasets with the same experimental conditions, attending to the left or right visual field (LVF or RVF), mimicking the modulation expected on the electrophysiological attentional correlates: contralateral P1/N1 complex and contralateral increase and ipsilateral increase of alpha, both in occipital channels.”

In the initial summary of the section 2.4. Data analysis, we have added a couple of sentences including the simulation analysis (page 10):

“Ultimately, we followed the opposite approach: we generated two datasets with the same experimental conditions as our empirical data (LVF and RVF), where underlying activity mimicked the modulations of attentional correlates, that is, ERPs and alpha. We then obtained their TGMs to reveal the pattern to expect under those electrophysiological conditions and asses the alignment between the empirical and simulated data.”

While the details of the data simulation are included in the following new section of the methods (pages 16, 17 and 18):

**“ 2.4.7. Data simulation**

Finally, we simulated two datasets with the same conditions of our empirical data, orienting attention to the left visual field (LVF) or to the right (RVF), to provide evidence of the relationship between the TGM pattern and the attentional correlates (i.e. occipital ERPs and alpha activity). The underlying brain activity between conditions systematically differed, following the attentional modulations in ERPs and alpha oscillations (Fig 5). This would grant us complete control over the ground truth brain patterns to generate TGMs which can then be compared with the empirical data.

To simulate the data with ERPs, we placed two homologous dipoles oscillating at 10 Hz in the visual cortex (in the left hemisphere to generate the RVF condition: x=-3, y=-93, z= 12, in the right hemisphere for the LVF condition: x=11, y=-92, z=12, see Fig 5A). Dipole orientation was mirrored for the two sources such that each diploe was oriented in opposing lateral directions (i.e. [-1 0 0] and [1 0 0] for left and right source, respectively). The dipole signal had a random phase across 60 trials during 4.6 s (time vector from -2 to 2.6 s, sampling rate 200 Hz). Then, we simulated a stimulus-induced phase reset: the phase of every trial shift to 0º at 70 ms, which was expected to result in a positive and negative peak between 100 and 150 ms after averaging, resembling the complex P1/N1. Eventually, a phase desynchronization was induced at 170 ms to prevent the ERP from continuing indefinitely (Fig 5B). We also injected the data with some variability to emulate natural brain dynamics (such as pink noise amplitude and frequency drifts). Using this model, the scalp distribution was generated at 128 channels (same as our empirical data). We used the ‘ft_dipolesimulation’ function from Fieldtrip [31]. We validated this simulated dataset by computing the average for each condition (LVF and RVF) and plotting the ipsilateral and contralateral ERPs of the same posterior electrodes used for the empirical data analysis (that is, P5/P6, P7/P8, P9/P10, PO7/PO8, PO9/PO10, PO11/PO12, I1/I2, POI1/POI2 and O1/O2). Lastly, we followed the same procedure described in section 2.4.2 to generate its TGM from these posterior channels.

**Fig 5. Simulated data.** A. Dipole location. This image shows both the location of the two dipoles used for the simulations (left and right visual cortex) and the electrodes on which the dipole signal was projected. B. ERP simulated data. The dipole signal of the dipole was used to generate each experimental condition is shown over three trials. It starts with a random phase, which is interrupted by a stimulus-induced phase reset. C. Alpha simulated data. This panel depicts one trial of the two dipole signals used to generate each cue condition (red and blue line). Both dipoles are in phase and, after the onset of the presumed cue at 0 ms, the contralateral one shows a power decrease (blue line) while the ipsilateral one increases in power (red line).

The second simulated dataset was intended to mimic a contralateral decrease and ipsilateral increase in occipital alpha power in relation to the attended hemifield after a cue onset (Fig 5C). Thus, two oscillating dipoles were placed on the left and right hemifield with a frequency of 10 Hz and a random phase across trials. In each experimental condition (LVF and RVF), the contralateral dipole signal initiated a power decrease at 0.35 s, peaking at 0.5-0.7 ms and reaching previous values at 1.25 s, meanwhile the ipsilateral dipole had a mirrored increase. All other parameters remained the same as in the ERPs simulation. The validation of this dataset consisted in the estimation of the oscillatory power using a Hanning-tapered sliding window Fourier transform. The data was decomposed in 10 ms steps, obtaining frequencies from 4 to 30 Hz in 1 Hz steps and with the Hanning window width adjusted to 5 cycles per frequency. Then, we plotted the posterior contralateral and ipsilateral alpha (10 Hz) for each condition. Eventually, we computed the TGM, using the posterior channels as features (see 2.4.2 section).

The last step was to determine which of the two simulated TGM aligns most closely with the empirical TGM obtained for each dataset. We computed a 2-D correlation coefficient for each participant correlating their empirical TGM with the two simulated TGMs (ERP and alpha). This was done separately for each dataset, resulting in a single correlation value per participant, simulation and dataset. Correlation coefficients were normalized by applying a Fisher Z-transformation and their absolute values were statistically tested with a two-factor ANOVA, with Simulation (ERP vs Alpha) as within subjects factor and Attentional Cue (peripheral vs central) as a between-subject factor. Greenhouse-Geisser correction was considered in case of nonsphericity and Bonferroni was used for multiple comparison correction. We estimated effect size using the partial eta-square (η_p_^2^) method. ”

Likewise, there is now a new heading in the results section (pages 26, 27, 28 and 29):

**“3.6. Simulation results**

Two simulated datasets with the electrophysiological correlates of attention (ERPs and alpha modulation) when attending to the LVF and RVF were generated to provide evidence on the TGM pattern resulting from that ground truth. In each dataset, the experimental conditions differed on the dipole location, which were placed in the right or/and left visual cortex (see Fig 5A).

The ERP simulation consisted of a single dipole oscillating at 10 Hz in the contralateral hemisphere to the simulated attentional cue, with a phase reset around 70 ms (see Fig 5B). The dipole orientation was reversed when moving the dipole to generate the other experimental condition. As expected, when computing the average across trials and conditions (LVF and RVF) to validate the dataset, two consecutive contralateral peaks were observed in the posterior electrodes (see Fig 10A, first row). Resembling the P1/N1 complex, the first component was positive and peaked at ≈100 ms, while the second one had a negative polarity and a ≈180 ms latency. Finally, the TGM computed on posterior channels showed a reverting pattern between 70 and 170 ms, coinciding with the ERP component latency (Fig 10A, second row). The higher accuracy values were found in the main diagonal, while the lowest ones spread off-diagonal, due to the P1/N1 polarity reversion between hemispheres and conditions.

**Fig 10 Simulated data.** A. ERP simulation. The first row depicts the average of the simulated data, for each cue condition and each posterior hemisphere. A contralateral P1/N1 complex can be observed (blue line), validating this data simulation. The second row is the TGM of this simulated dataset, revealing an early reverting pattern at the P1/N1 complex latency. B. Alpha simulation. In the first row, the 10 Hz power of the simulated data has been displayed, again for each cue and posterior hemisphere. The blue line shows the expected contralateral alpha decrease. The second row depicts the TGM, where no pattern can be observed.

Regarding the alpha simulation, both experimental conditions had one 10 Hz oscillating dipole in each hemisphere. There was a power modulation from 0.35 to 1.2 s (Fig 5B), with a power increase in the ipsilateral dipole (red line) and a power decrease in the contralateral one (blue line). This modulation can be observed when decomposing the simulated signal into frequencies: posterior contralateral channels showed a decrease at 10 Hz during the same latency (Fig 10B, first row). However, this neural signature was not informative for the classifier to be able to decode the two experimental conditions, as shown by the TGM with random accuracy values (Fig 10B, second row).

Eventually, we computed the correlation between the empirical and the simulated TGMs and applied a two-way ANOVA with Simulation (ERP or alpha) as a within-subjects factor and Attentional Cue (peripheral cue dataset and central-symbolic cue dataset) as a between-subject factor to assess which simulation best fits the empirical datasets. This test revealed significant main effects for Simulation (F_1_=8.46, p=.006, η_p_^2^=0.15, β-1=.81) and Attentional Cue (F_1_=5.54, p=.023, η_p_^2^=0.11, β-1=.64) and a significant interaction between both factors (F_1_=4.62, p=.037, η_p_^2^=0.09, β-1=.56). Specifically, when considering the best fit for the peripheral cue dataset (see Fig 11, left side), higher correlations were observed with the ERP simulation (M=.030, SD=.005) than with the alpha simulation (M=.012, SD=.002, t_(23)_=2. 28, p<.001), but no differences were found in the central-symbolic cue dataset (see Fig 11, right side). Additionally, the coefficients obtained from correlating each dataset with the ERP simulation (see green data in Fig 11) were higher for the peripheral cue dataset (M=.030, SD=.005) than for the central-symbolic cue dataset (M=.014, SD=.004, t_(47)_=2.48, p=.017), while there were no differences in the alpha simulation (red data in Fig 11).

**Fig 11 Empirical data and simulation alignment.** After obtaining the 2-D correlation coefficient between each participant TGM for the peripheral and central cue datasets (left and right, respectively) and each simulated TGM (EPR - green and alpha - red), the absolute values of its Fisher z-transformation have been represented on this raincloud plot. Crosses represent outliers.”

These results have been integrated with the ideas previously discussed (page 33):

“Importantly, the simulated data provide insight into the debate regarding the underlying brain activity that leads to specific TGM patterns. Our ERP simulated data shows that a stimulus-induced phase reset, or in other words, an ERP, simultaneously in different hemispheres can be decoded by a classifier. This is our speculation regarding the empirical peripheral dataset, where both the ERP modulation and the simultaneous reverting TGM pattern can be observed, with a significant correlation (Figure 7). However, there is not such a reversion in the TGM of the central cue dataset, where we controlled for purely sensory-driven processes related to the cue onset. Thus, although the P1/N1 component is evident (Fig 8), the classifier result suggests that it must have different characteristics, as there is no successful decoding. However, the scenario in relation to alpha is simpler, whose simulation leads to the same results as both empirical data: the power modulation in alpha is not detectable by the classifier in order to distinguish both experimental conditions (see Figure 7 and 8). Indeed, our analysis of the fit between the simulated and empirical data points out that the peripheral cue aligns better with the ERP simulation compared to the alpha simulation, supporting the idea that ERP activity drives the observed pattern. Therefore, our simulations support the conclusions drawn by relating the electrophysiological correlates of attention to the classifier performance.”

Eventually, the abstract and conclusions have been updated accordingly (see page 2 and 35).

**The analyses aimed at ruling out the effects of ERPs and Alpha on classification accuracy are another point of concern. The methods used are not optimal given the different transformations applied to these signals. I recommend using sliding window analyses and/or cross-correlation techniques to ensure a more robust interpretation of the data and to disentangle the contributions of these factors more convincingly.**
Response: we thank the reviewer for suggesting this additional contrast. We believe a cross-correlation should indeed be helpful to reveal periodicities between ERPs and alpha and the classification accuracy. Therefore, we have included it in our analysis, as a previous step to the correlation we had already provided:

“First, we used cross-correlations, not only to determine how well these measures match each other and at what point, but also to reveal possible periodicities previously described in our hypothetical scenarios of attentional sampling (see Fig 1). Thus, we correlated the grand average of ERPs and alpha-band LI with the average TGM diagonal accuracy in the analyzed temporal window formerly analyzed, sliding the data every time sample. The obtained results were normalized to get indices bounded between -1 and 1 and its absolute values were depicted (as the sign simply depends on the order of subtraction in the LI calculation, that is left cue-right cue or right cue-left cue).

The cross-correlation results revealed that the best match between the time series was around 0ms-lag (see results section), so we subsequently computed the correlation coefficient between TGM diagonal accuracy and ERPs/alpha-band LI, timepoint by timepoint along the orienting period over participants” (in page 15, section 2.5.6. Lateralization Index Classifier Accuracy Correlation).

The highest cross-correlation values were around the 0ms-lags in both datasets 1 and 2 and for both ERPs and alpha power, plots which have been included as the third row of Figures 8 and 9. Find below the figures and the changed section of the manuscript corresponding to the description of these new results (pages 23, 24, 25 and 26):

“ **3.4. ERPs and alpha results for dataset 1 (peripheral cue)**

To find out whether the neural correlates of attention as measured with ERPs and alpha power are related to the classifier performance, we calculated a lateralization index (LI), independently for occipital ERPs and alpha-band power. These LIs were then cross-correlated and correlated with the accuracy values of the TGM’s main diagonal independently for dataset 1 and dataset 2, shown in Figs 8 and 9 respectively, with ERPs depicted in the left panel and alpha-band power in the right panel.

Cue stimulation in dataset 1 elicited three ERP components: P1, N1 and P2 (see top image of the left panel in Fig 8). The LI, calculated by subtracting ipsilateral from contralateral activity, shows different peaks whose polarity changes coincide with the alternating yellow and blue blobs in each row of the TGM (see second image of the left panel in Fig 8). However, the cross-correlation does not have an alternating pattern matching the lag between peaks, but a sustained decreased between -125 and 215 ms-lags (third image of the left panel in Fig 8). Indeed, the correlation time point by time point between the LI and the TGM diagonal resulted in significant correlations between ~100 to ~650 ms, which includes both the early and the late TGM blobs (see bottom image of the left panel in Fig 8).

**Fig 8. Conventional univariate analysis for dataset 1 (peripheral cue).** Occipital ERPs and alpha-band activity are shown in the left and right panel respectively. The upper row depicts the lateralized activity, blue line for ipsilateral and red line for contralateral, averaged for left and right cue conditions. In the second row, the lateralization index (LI: contralateral – ipsilateral activity) is shown overlaid on the average TGM. The third row displays the cross-correlation between TGM diagonal accuracy and LI. The bottom row shows the correlation coefficient between the same measures. The orange dots highlight the time points where the correlation is significant (cluster corrected).

Concerning alpha-band power a pronounced power decrease for both ipsilateral and contralateral activity after the cue onset was observed in dataset 1 (see top image of the right panel in Fig 8). The LI, second image, shows a higher decrement for the contralateral alpha in the vast majority of the orienting period. Again, the cross-correlation exhibits a negative sustained pattern around 0 ms-lags (from -100 to 60 ms-lags, see third image if the right panel in Fig ), but with smaller values. However, although the LI waveform appears to overlap with some TGM patterns no strong continuous correlations between alpha power LI and TGM diagonal were observed (bottom image of the right panel in Fig 8).

3.5. **ERPs and alpha results for dataset 2 (central-symbolic cue)**

The symbolic retro-cue used in dataset 2 elicited the same three ERP components, that is, P1, N1 and P2 (Fig 9, top image of the left panel). However, LI did not seem to overlap with the accuracy changes of the TGM (see second image of the left panel in Fig 9). The cross-correlation values slightly fluctuate between 0 and -5. However, the maximum of the temporally shifted correlations can be found with a 0 lag, which is in line with the lack of significant results in the correlation between LI and the TGM diagonal (bottom right panel in Fig 9).

**Fig 9. Conventional univariate analysis for dataset 2 (central-symbolic cue).** Occipital ERPs and alpha-band activity are shown in the left and right panel respectively. The upper row depicts the lateralized activity, blue line for ipsilateral and red line for contralateral, averaged for left and right cue conditions. In the second row, the lateralization index (LI: contralateral – ipsilateral activity) is shown on top of the average TGM. The third row displays the cross-correlation between TGM diagonal accuracy and LI. The bottom row shows the correlation coefficient between the same measures. The orange dots highlight the time points where the correlation is significant (cluster corrected).

Ipsilateral and contralateral alpha-band power is observed after the cue onset in the dataset 2 (Fig 9, right panel, top image). The LI, in the second raw, reflects a contralateral alpha decrease from ~600 ms, which coincides with the high accuracy blob of the TGM during several milliseconds. This also matches the cross-correlation sequence (third image in the right panel of the Fig 8), where the values abruptly drops from 0 to ~ -10 between 0 and 340 ms-lags, pointing to an stable attentional sampling. However, the correlation coefficient only shows isolated significant time points between alpha power LI and TGM diagonal at the beginning of the orienting period (bottom image in the right panel, Fig 9).”

In summary, the main pattern is a 200-300ms sustained negativity which can only account for a stable and simultaneous mechanism between both measures. Therefore, the most informative contrast is the classical correlation initially provided, computed time point by time point between ERPs/alpha and the classifier accuracy. This is also mentioned now in the conclusion section: “Particularly, we were looking for a periodicity-sensitive prediction measure, so we used a cross-correlation. However, a steady decreasing pattern around 0ms-lag was systematically found, making the time point by time point correlation coefficient the most informative measure.” (page 32, third paragraph).

**I would also like to understand why the authors emphasize the distinction between conventional analyses and MVPA so strongly. As it stands, the manuscript seems to introduce more confusion about potential processes at play rather than providing clear insights into the underlying physiology.**

Response: We thank the reviewer for pointing out the lack of clarity here. Emphasizing the distinction between conventional analysis and multivariate approaches is particularly relevant because this topic has traditionally been addressed with conventional analysis. This is now explicit in the Introduction section: “Thus, we combined the analysis traditionally employed on this topic, ERPs and time-frequency (TF) analysis, together with a time-sensitive decoding approach that tracks the location of attention on a millisecond-by-millisecond basis in parieto-occipital electrodes during an informative cueing paradigm” (page 4, first paragraph).

Certainly, our results do not reveal what the underlying neural mechanisms of the attentional sampling are. However, the data points strongly to the existence of an occipital attentional spotlight that tracks consistently both visual hemifields.

**Moreover, there appears to be insufficient effort to link these findings to behavior. Identifying differences in classification accuracy is not equivalent to uncovering a physiological process; it is merely another statistical approach. Bridging these analytical results with behavioral outcomes could significantly enhance the study’s impact and relevance.**

Response: Thank you for raising this important issue. The initial goal of this study was to obtain neural evidence of the attentional dynamics using a novel multivariate approach. We used time generalization matrices (TGM), whose computation requires trials with the same time length. Unfortunately, this is incompatible with the collection of useful data at the behavioral dimension. As others have already done (Fiebelkorn et al., 2013a; Landau & Fries, 2012; VanRullen et al., 2007), trials with variable orienting period are essential to properly explore the behavioural dynamics of attention, which would interfere with the neural analysis approach. Additionally, this manipulation would have required increasing considerably the number of trials to have enough observations at the neural level (cue conditions x orienting period lengths). These limitations are now explicit in the discussion section: “That is, we would need trials with different time length, as opposed to those required for the TGM computation. Furthermore, this would imply increasing considerably the number of trials, in order to have enough observations at neural level (cue condition x orienting period lenght).” (page 34, second paragraph).

**Finally, the introduction mentions a role of prefrontal and parietal regions. Why is this spatial diversity not represented in the analysis, using for instance spotlight analysis and whole-scalp decoding, to explore in more detail whether other areas might be relevant to the process the author seem to want to explore.**

Response: We thank the reviewer for pointing out the lack of clarity here. The reason for focusing our analysis on occipital electrodes is the role of occipital areas as responsible for visual processing. At this very early stage of the visual system, EEG recordings show traditionally contralateralized spatial processing (ERPs and alpha activity). Thus, we expected to be able to observe the attentional visual sampling in these channels. Meanwhile, the parietal lobe does not carry information about the locus of attention, but it is involved in attentional orientation deployment (Posner & Dehaene, 1994). In other words, it shows similar activity across both classes (i.e. LVF vs RVF) and therefore would not be picked up by the classifier as employed here. Finally, the prefrontal cortex has been described as the origin of the attentional control signal (Buschman & Miller, 2007; Gregoriou et al., 2014; Moore & Fallah, 2004), in particular, the right ventral prefrontal cortex has been related to inhibitory control (Aron et al, 2003; Chambers et al, 2007). Note that the studies that find the involvement of these different regions in attention allocation use local recordings in non-human primates (see page 3, “Overall, evidence mainly comes from local recordings of prefrontal and parietal areas in non-human primates, although a few studies have shown fluctuations also percolate visual regions [21-22]” or page 32 second paragraph “Previous studies have found that visual areas, frontal eye-fields and lateral intraparietal sulcus are involved in attention allocation, using local recordings in non-human primates in a hypothesis-driven manner [16, 20-22]. Like these studies, we used specific EEG channels based on the occipito-parietal origin of the attentional correlates [4, 8-9]. However, our recordings are from human volunteers and we employed a data-driven approach (i.e., MVPA) to analyse brain activity.”). This does not guarantee that we will be able to see this activity in human scalp recordings. Therefore, we focused our analysis on occipital electrodes.

However, to answer the reviewer’s curiosity, we have computed the grand average TGM training the classifier on the full set of 128 electrodes and significantly tested for both datasets. We compare this result with the occipital TGM already included in the manuscript in the following figure:

Apparently, training the classifier using 128 channels (left column) or the occipital channels (middle column) results in similar TGM patterns, but with higher accuracy values for the occipital electrodes. Interestingly, the statistical test shows that the cluster from the occipital channels (black mask) overlaps with most of the 128 electrodes cluster (brown mask) for the first dataset (upper row). Only an earlier window, 400-500ms along the diagonal, spreading to off-diagonal areas, is exclusively decoded when taking 128 channels (beige-coloured mask). In the case of the second dataset, the classifier can even decode significantly a larger area when the information comes from occipital channels (black mask), in off-diagonal time points of the brown and beige-coloured areas, but also in later time points where the 128 channels significant mask does not reach (800-1,200ms).

Therefore, extending our analysis to the full set of electrodes would mean losing decoding accuracy and, thus, information about the occipital spotlight of attention.

**In summary, while I believe the approaches employed in this paper have potential, more work is needed to unify these methods and present a cohesive narrative. Strengthening the link between the proposed models, the analytical results, and behavioral evidence would greatly improve the clarity and significance of the findings.**

Response: We would like to thank the reviewer for the positive comments and the valuable feedback which helped us to improve our manuscript.

**Minor comments:
-The layout of figure 5 is unconventional (i.e. the order of A, B, C, D) and it is badly mentioned on page 18**

Response: Thank you for pointing out these typos. The figure and the text have been corrected (pages 19, 20, 21 and 22, 3.6. Classification results for dataset 1 (peripheral cues) section).

**- The intro mentions ‘recent finding’ but then cites papers from 2013, 2012, 2007, to me, only the critical review seems recent**

Response: Thank you for noticing, we have replaced the adjective “recent” by “subsequent” (Introduction, page 3, first paragraph and Discussion, page 30, second paragraph).
